# RRHP: a tag-based approach for 5-hydroxymethylcytosine mapping at single-site resolution

**DOI:** 10.1186/s13059-014-0456-5

**Published:** 2014-09-24

**Authors:** Adam Petterson, Tzu Hung Chung, Darany Tan, Xueguang Sun, Xi-Yu Jia

**Affiliations:** Zymo Research Corporation, 17062 Murphy Ave., Irvine, CA 92614 USA; Current address: 5200 Illumina Way, La Jolla, 92122 USA

## Abstract

Current methods for genomic mapping of 5-hydroxymethylcytosine (5hmC) have been limited by either costly sequencing depth, high DNA input, or lack of single-base resolution. We present an approach called Reduced Representation 5-Hydroxymethylcytosine Profiling (RRHP) to map 5hmC sites at single-base resolution by exploiting the use of beta-glucosyltransferase to inhibit enzymatic digestion at the junction where adapters are ligated to a genomic library. Therefore, only library fragments presenting glucosylated 5hmC residues at the junction are sequenced. RRHP can detect sites with low 5hmC abundance, and when combined with RRBS data, 5-methylcytosine and 5-hydroxymethylcytosine can be compared at a specific site.

**Electronic supplementary material:**

The online version of this article (doi:10.1186/s13059-014-0456-5) contains supplementary material, which is available to authorized users.

## Background

Since 2009, one of the most rapidly developing subdisciplines in molecular genetics has proven to be the identification and characterization of 5-hydroxymethylcytosine (5hmC). Like its close relative, 5-methylcytosine (5mC), 5hmC is one of the covalent modifications observed in prokaryotic and eukaryotic genomes [1,2], which constitute an important class of epigenetic modifications. At present, the precise role of 5hmC in the genomic context is under close study from a myriad of angles. One paradigm implicates 5hmC in the oxidative demethylation of cytosine [3], which has been bolstered by subsequent characterizations of 5-formylcytosine (5fC) and 5-carboxylcytosine (5caC) in the genome [4]. Aside from its mechanistic characterization, 5hmC localization and tissue distributions have also been extensively studied, resulting in clear demonstration of elevated abundance in tissues within the central nervous system (CNS) [5]. Pathologically, a profound depletion of 5hmC is observed across several malignant carcinomas [6].

As a result of the increased study of this modification, more sensitive tools are required for detection, quantitation, and ultimate mapping of the marker across the genome. While methods such as LC-MS/MS-MRM are useful for sensitive detection and quantitation of 5hmC and other modified nucleosides, most genetic applications require the ability to pin down the mark to a tight region, locus, or specific junction within a locus. Several technologies have become available that utilize old methodologies, such as immunoprecipitation or qPCR, as well as new methodologies, including chemical labeling, single-molecule kinetic monitoring, nanopore conductivity, and more. A recent review highlights the advantages and pitfalls of these techniques [7].

For many applications, genome-wide approaches, including hMeDIP and bio-orthogonal labeling with glucosylation, provide robust enrichment pools for Sanger sequencing as well as massively parallel (next-generation) sequencing. Despite good coverage of the genome and high specificities, these methods are often limited by input requirements which typically are in the neighborhoods of several micrograms. These amounts of DNA are often not feasible for investigation of precious samples such as stem cells or selectively isolated cellular subpopulations (that is, diverse neuronal cells from a whole brain sample). Importantly, these enrichment-based methodologies, even in highly optimized protocols, lack single-base resolution, and identified hydroxymethylated sites will fall within the range of several hundred to several thousand bases. Depending on how well a particular region is annotated, such resolution is often insufficient to describe activity in transcriptionally relevant sites with confidence. Findings from such studies require subsequent validations with locus-specific assays, such as glucMS-qPCR, to enhance the 5hmC positions.

Recently, two approaches which enable quantitative, single-base resolution mapping of 5hmC have been reported. Oxidative bisulfite sequencing (oxBS-Seq) [8] takes advantage of selective chemical oxidation via organometallic catalysis to yield 5fC from 5hmC, which is then susceptible to traditional bisulfite conversion and results in a different sequencing signal from the 5mC sibling. The 5hmC level is inferred by comparing the methylation values between the modified and traditional bisulfite sequencing. Although this process allows interrogating of 5hmC at single-base resolution, the oxidation step leads to significant DNA degradation (approximately 0.5% of original DNA fragments are retained through the process, according to the authors), which again restricts its application to very rare samples. In addition to this approach, great strides have been reported with the Tet-assisted Bisulfite Sequencing (TAB-Seq) [9] approach. In this methodology, 5hmC positions are initially protected by glucosylation and then treated with the Tet enzyme to selectively oxidize naked 5mC positions to 5hmC and then 5fC and 5caC. These 5fC or 5caC positions are susceptible to bisulfite conversion and deamination, so the only remaining cytosine positions are those originating from 5hmC. While the method avoids harsh organometallic treatment for oxidation, it extensively depends upon the Tet enzyme, which is known to present low efficiency (the authors suggested an efficiency of ≥90%, which can render at least 10% of methylated residues unconverted) [9]. Unconverted positions would, therefore, be falsely identified as 5hmC sites and contribute to a higher background signal for the assay.

As an alternative to these methods, we present a novel approach, known as reduced representation 5-hydroxymethylcytosine profiling (RRHP), that avoids harsh chemical conversion processes and affords sequence-level resolution of 5hmC positions. The method features a rapid workflow (<24 total h), allows for starting inputs as low as 100 ng, and offers strand-specific information about 5hmC distribution. The absence of chemical conversions also allows for sequencing of native DNA sequences, which enhances sequencing quality and resulting mapping ratios. Most importantly, the method proves to be a highly reproducible, positive display method, allowing for higher confidence when interrogating positions with low 5hmC content. When combined with existing reduced representation bisulfite sequencing (RRBS) data for the same sample, RRHP allows for both high resolution and accurate quantitation of 5mC and 5hmC positions across the genome simultaneously.

## Results

### Preparatory scheme and initial evaluation

**Figure 1:**
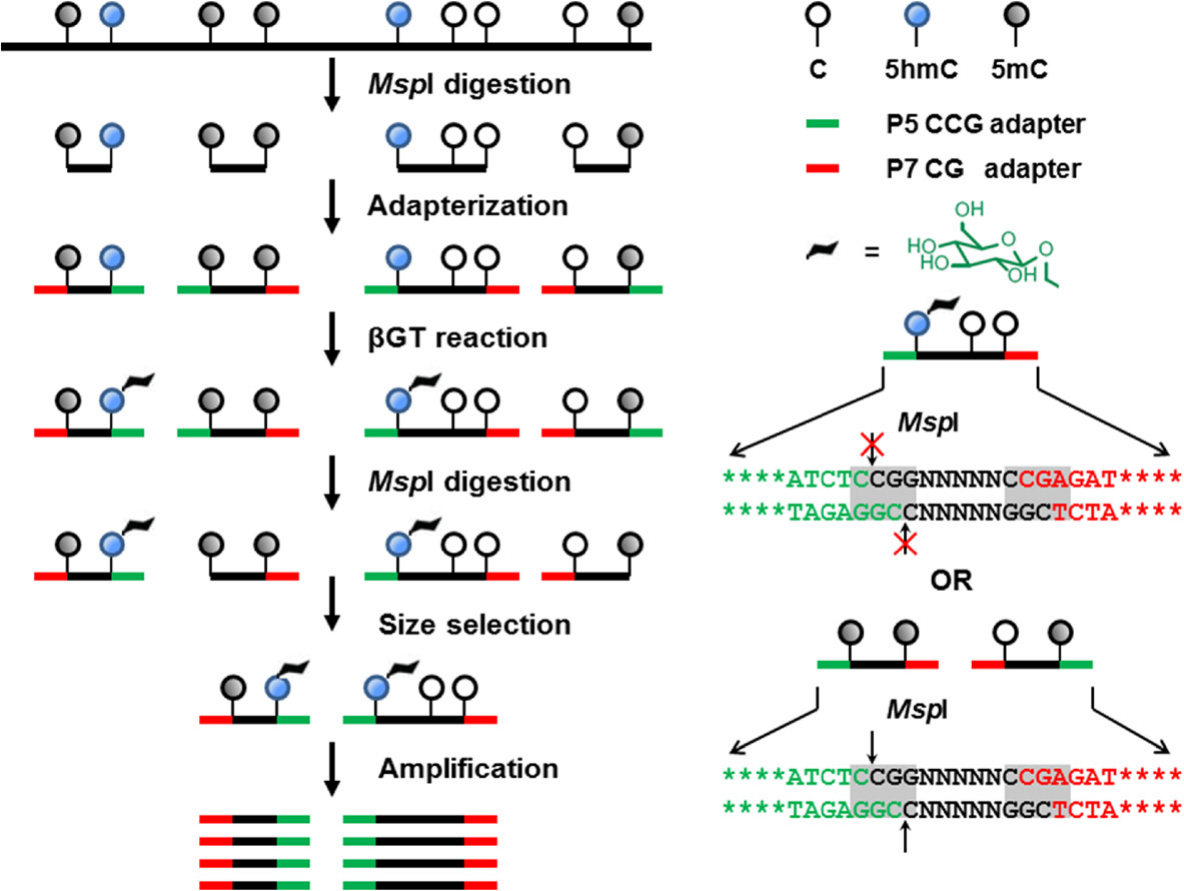
**Workflow of the RRHP assay.** Genomic DNA was first digested by restriction endonuclease MspI and then ligated with the modified adapters such that the MspI site is reconstituted at the junction of P5 adapter and fragment but not at the junction of P7 and fragment. The adapter-ligated fragments are either homo- or hetero-adapterized. Homo-adapterized fragments have the same adapters at both ends (P5-P5 or P7-P7), which will form an inhibitory stem-loop hairpin preventing itself from further amplification, whereas the hetero-adapterized fragments have different adapters (P5-P7). All ligated library fragments are glucosylated with β-glucosyltransferase (β-GT). Fragments presenting 5hmC at the junction and, thus glucosylated, are resistant to a second MspI digestion while fragments with unmodified C or 5mC are cleaved, removing the P5 adapter. Only the fragments with both P5 and P7 adapters after the second MspI digestion will be amplified and sequenced.

RRHP is dependent upon the availability of restriction endonucleases that exhibit insensitivity to methylation or hydroxymethylation within their cut sites while maintaining sensitivity to glucosyl modifications. In the simplest embodiment of the method, we digested human cerebellum DNA with MspI (which cleaves the CˇCGˆG pattern, regardless of the 5mC or 5hmC status) and ligated the resulting fragments to modified adapters compatible with the Illumina TruSeq P5/P7 series. The adapters were designed such that the MspI site was reconstituted at the junction of the P5 adapter and DNA fragment but not at the junction of the P7 adapter and DNA fragment. 5hmC within the ligated library fragments were glucosylated with β-glucosyltransferase (β-GT). Fragments with a glycosylated 5hmC at the junction were resistant to a second MspI digestion while fragments with an unmodified C or a 5mC were cleaved, therefore, removing the P5 adapter and preventing the library fragments from being amplified. Following size selection and limited amplification, the libraries were sequenced using the standard Illumina TruSeq workflow without any modifications to the standard configurations or reagents (Figure 1). Each sequencing read with a CCGG tag at the beginning of the read represented one 5hmC site.

**Figure 2:**
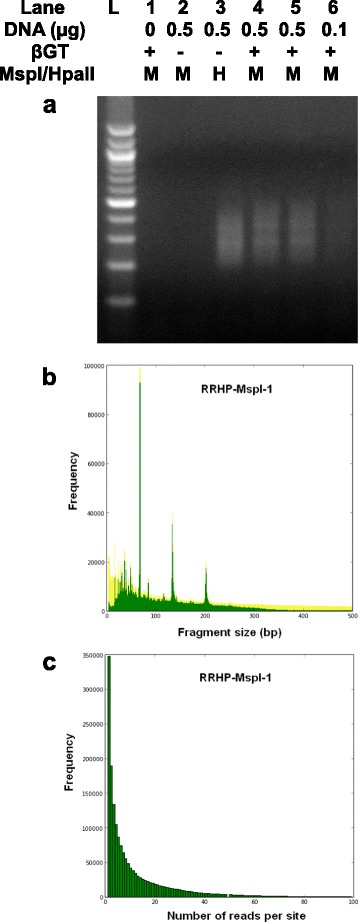
**Library and sequencing characteristics of RRHP. (a)** RRHP libraries prepared with different DNA input, with (+) or without (-) βGT, were digested by either MspI (M) or HpaII (H). L is 100 bp Marker (Zymo Research). **(b)** MspI digested fragments (green) detected by the RRHP assay overlaid with the fragments (yellow) generated from an *in silico* simulation. **(c)** The frequency distribution of 5hmC sites with different read coverage profiled by the RRHP assay.

To evaluate the method, we prepared six libraries (Figure 2a). Two negative control libraries (lane 1: without DNA input, lane 2: without the glucosylation step) demonstrated no observable product during the final amplification. Two duplicate libraries with 500 ng DNA input (lane 4 and 5) gave product within the expected range, as did the library from 100 ng DNA input (lane 6). We also prepared a library (lane 3) where the final cleavage was performed with HpaII, a methylation-sensitive isoschizomer, instead of MspI. This alternative digestion scheme allowed for the identification of any form of methylation at the adapter-fragment junction. A RRBS library [10] was also generated from the same brain sample for parallel comparison.

### Sequencing analysis

**Figure 3:**
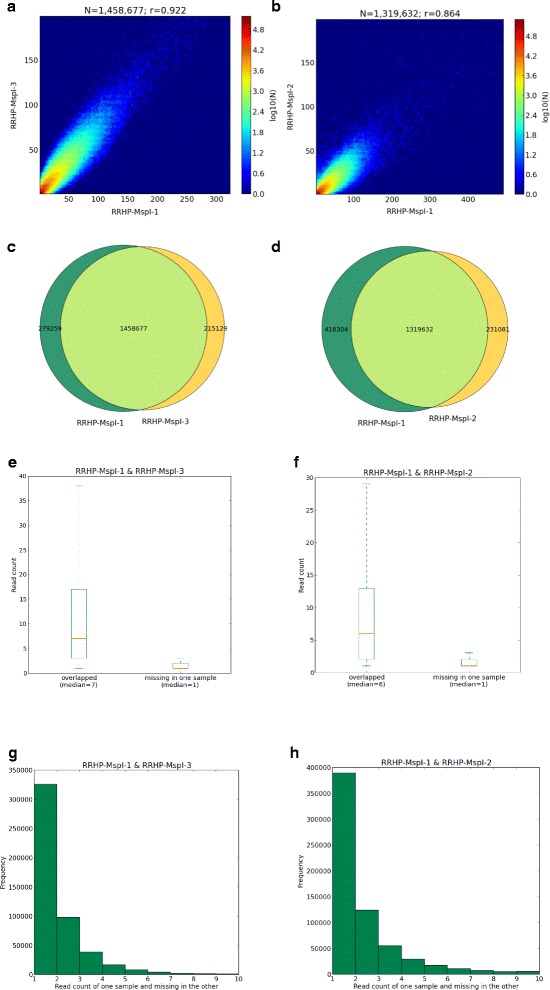
**Analysis of RRHP libraries prepared with different DNA input and sequencing depth. (a)** Scatter plot of the read counts between RRHP-MspI-1 and RRHP-MspI-3. **(b)** Scatter plot of the read counts between RRHP-MspI-1 and RRHP-MspI-2. **(c)** Venn diagrams show RRHP-MspI-1 and RRHP-MspI-3 have 1.45 million 5hmC sites in common while there are 279,259 unique sites in RRHP-MspI-1 and 215,129 unique sites in RRHP-MspI-3. **(d)** Venn diagrams show RRHP-MspI-1 and RRHP-MspI-2 have 1.3 million sites in common while there are 418,304 unique sites in RRHP-MspI-1 and 231,081 unique sites in RRHP-MspI-3. **(e)** Box plots shows that 5hmC sites overlapping in RRHP-MspI-1 and RRHP-MspI-3 have higher read counts on average than non-overlapping sites. **(f)** Box plot shows 5hmC sites overlapping in RRHP-MspI-1 and RRHP-MspI-2 have higher read counts on average than non-overlapping sites. **(g)** Distribution of read counts of non-overlapping 5hmC sites between RRHP-MspI-1 and RRHP-MspI-3. **(h)** Distribution of read counts of non-overlapping 5hmC sites between RRHP-MspI-1 and RRHP-MspI-2.

We obtained 23 million reads for the RRHP library prepared from 500 ng DNA input of which 95% mapped to the reference genome and 95% of the mapped reads demonstrated a CCGG tag at the start of the read. In total, 1.74 million unique MspI sites were profiled, accounting for 94.3% of detectable MspI sites within fragments selected from 40 to 430 bp from an *in silico* digestion (Table 1). We observed high concordance in fragment distribution by overlaying experimental data with the simulation (Figure 2b). A large number of 5hmC sites (44%) profiled were covered by less than five CCGG-tagged reads (Figure 2c and Additional file 1); since read number is proportional to 5hmC display, this indicated detection of 5hmC sites with relatively low abundance. We compared the HpaII-digested and MspI-digested libraries and observed that 85.6% of 5mC sites overlapped with 5hmC sites. Our results from RRHP agreed with observations from a TAB-sequencing study and supported the notion that 5hmC is ubiquitous in the genome but with a lower abundance than the 5mC mark [9]. Pairwise comparison between MspI libraries prepared with different DNA inputs (RRHP-MspI-1 and RRHP-MspI-3) showed a high concordance (Pearson’s coefficient =0.92), and nearly 1.46 million 5hmC sites (83.3%) overlapped between the two libraries (Figure 3a and 3c). To determine the effects of read depth on detection sensitivity, we sequenced one of the duplicated libraries (RRHP-MspI-2) with 50% less depth and obtained roughly 10 million reads with similar mappability (94.9%). Correlation between the replicates was lower than the samples with a higher read depth, but the Pearson’s coefficient was still 0.86 and 1.32 million sites (75% of the total profiled sites) overlapped in both libraries (Figure 3b and 3d). Further analysis showed that the non-overlapped sites between the two libraries had lower read counts compared with the overlapped sites (Figure 3e and 3f), and about 95% of these sites had a read count below three. This indicated that higher sequencing depth is needed to enhance the reproducibility and to confirm the presence of 5hmC with low abundance.

**Table 1:** **Statistical analysis of sequencing reads from** ***in silico*** **simulation and RRHP experiment**

	**Simulation**	**RRHP-HpaII**	**RRHP-MspI-1**	**RRHP-MspI-2**	**RRHP-MspI-3**	**RRHP**	**RRBS**
	**40-430 bp**	**(0.5 μg)**	**(0.5 μg)**	**(0.5 μg)**	**(0.1 μg)**	**(- βgt control )**	
Total reads	1,845,334	23,702,341	23,383,403	9,505,230	19,539,657	5,518	41,066,513
Mapped reads	1,845,025	20,605,538	22,271,499	9,017,016	18,482,119	4,373	15,668,523
Mappability (%)	99.98	86.93	95.24	94.86	94.59	79.25	38.15
Tagged reads (n)	1,845,025	17,763,501	21,081,749	8,531,903	17,328,911	3,230	NA
Tagged reads (%)	100	86	95	95	94	74	NA
5hmc sites (n)	1,845,014	1,878,394	1,737,993	1,550,791	1,674,080	3,171	5,330,488

By comparing the data from RRHP with RRBS, we were also able to estimate the minimal read counts needed to reach <5% error rate in 5hmC detection. Since both RRHP and RRBS employ MspI for fragmentation during library preparation, we were able to simultaneously compare 5hmC with 5mC for certain sites. In principle, any CpG site with zero methylation in RRBS should not have any reads in RRHP. However, if there is a RRHP read detected in those sites, it could have resulted from a false calling. Based on that, we calculated the error rate for RRHP sites with various read counts by selecting CpG sites from RRBS with greater than 50× read coverage and zero methylation and cross-checking them with MspI cutting sites (CCGG) which were considered as potential detectable RRHP sites. In total, 1,635 CpG sites passed the criteria and this number was denoted as N. Any CpG site with i reads (i ≥1) from the RRHP assay was considered as a false hydroxymethylated site. The error rate was calculated as Error rate (E_i_) = Number of N_i_/Number of N. As shown in Table 2, the false calling rate was less than 5% when read counts reached four or greater. If we exclude 5hmC sites with a low read coverage (<5) to remove the noise introduced by spurious reads, interestingly, the percentage of overlapping sites did not change significantly between the technical replicates with the same sequencing depth (Additional file 2A). However, the correlation metrics increased constantly with the higher read cutoff (Additional file 2B). Due to the positive display nature of RRHP, we also observed a substantial overlap of 5hmC positions only within the methylated sites detected by RRBS (Additional file 3).

**Table 2:** **Calculated false calling rates of RRHP for various read counts**

**Read cutoff no.**	**RRHP-MspI-1**		**RRHP-MspI-2**		**RRHP-MspI-3**	
	**Error rate**	**Error count**	**Error rate**	**Error count**	**Error rate**	**Error count**
1	0.358	585	0.474	775	0.242	396
2	0.143	233	0.191	312	0.106	173
3	0.057	93	0.079	129	0.054	88
4^a^	0.026	42	0.028	46	0.029	48
5	0.02	32	0.013	21	0.02	33
6	0.015	24	0.007	11	0.015	25
7	0.009	15	0.004	7	0.011	18
8	0.008	13	0.004	7	0.008	13
9	0.006	9	0.004	6	0.006	10
10	0.005	8	0.003	5	0.004	7

^a^Minimal read counts required for an error rate <0.05.

### Annotative characteristics and unique features

**Figure 4:**
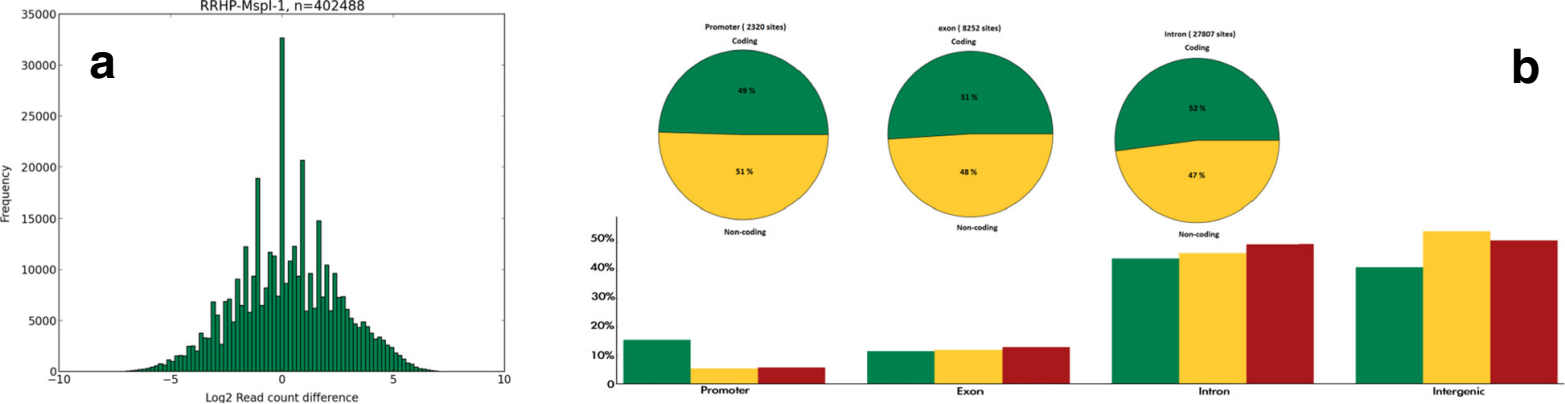
**5hmC is asymmetrically distributed between the forward and reverse strand throughout the genome. (a)** Distribution of the read count difference (log2 scale) between the forward and reverse strand for sample RRHP-MspI-1. **(b)** CpG sites with asymmetric 5hmC distribution between the forward and reverse strand were analyzed according to gene annotation (column plot) and coding (pie chart). Legend for annotation column plot - Green: detectable MspI sites; Yellow: MspI site with 5hmC detected from both strands; Red: MspI sites with two-fold 5hmC difference between the strands. Legend for pie chart - Green: coding strand; Yellow: non-coding strand.

Functional annotation analysis of our RRHP data (Additional file 4) revealed a large number of 5hmC sites profiled were located within annotated genes, especially introns (45%), 5′UTR (12%), and 3′UTR (5%). Interestingly, only 10% of the total 5hmC sites profiled by RRHP overlapped with CpG islands despite the intrinsic bias of the assay towards high CpG densities; nevertheless, 88% of the CpG islands of the entire genome were covered by at least one 5hmC site. Another 8% of 5hmC sites were mapped to promoters, predominately in high CpG promoters (HCP), and 82% of gene promoters were covered by at least one 5hmC site. We also observed overlap of 5hmC sites to diverse regulatory elements such as histone methylation, indicating broad survey of regions other than CpG islands and promoters. To examine whether 5hmC sites are associated with genes of specific functions, we performed gene ontology analysis using GREAT [11], which allows functional analysis of *cis*-regulatory regions such as enhancers. Interestingly, no significant gene enrichment of any cellular processes was found to be associated with 5hmC. The RRHP assay can also detect the strand distribution of 5hmC because library construction is directionally unbiased. By counting the number of reads with the CCGG junction generated at the sense or antisense strand, we can determine their respective 5hmC abundance. Consistent with previous observations [9], a large number of CpG sites showed an asymmetric distribution of 5hmC (Figure 4A). About 50,000 sites were found to have read counts with at least a two-fold difference between the strands. Further analysis indicated there were no obvious preference of 5hmC to either the coding strand or different annotated regions such as promoter, intron, exon, and so on, indicating that the distribution of 5hmC are random between the stands (Figure 4B). In addition, since library preparation does not involve bisulfite conversion, we can directly identify single nucleotide polymorphisms (SNPs) within reads with high confidence (Additional file 5). Thus, both genetic variations and epigenetic modifications can be analyzed from a single data set.

### Cross-platform validations and correlation

**Figure 5:**
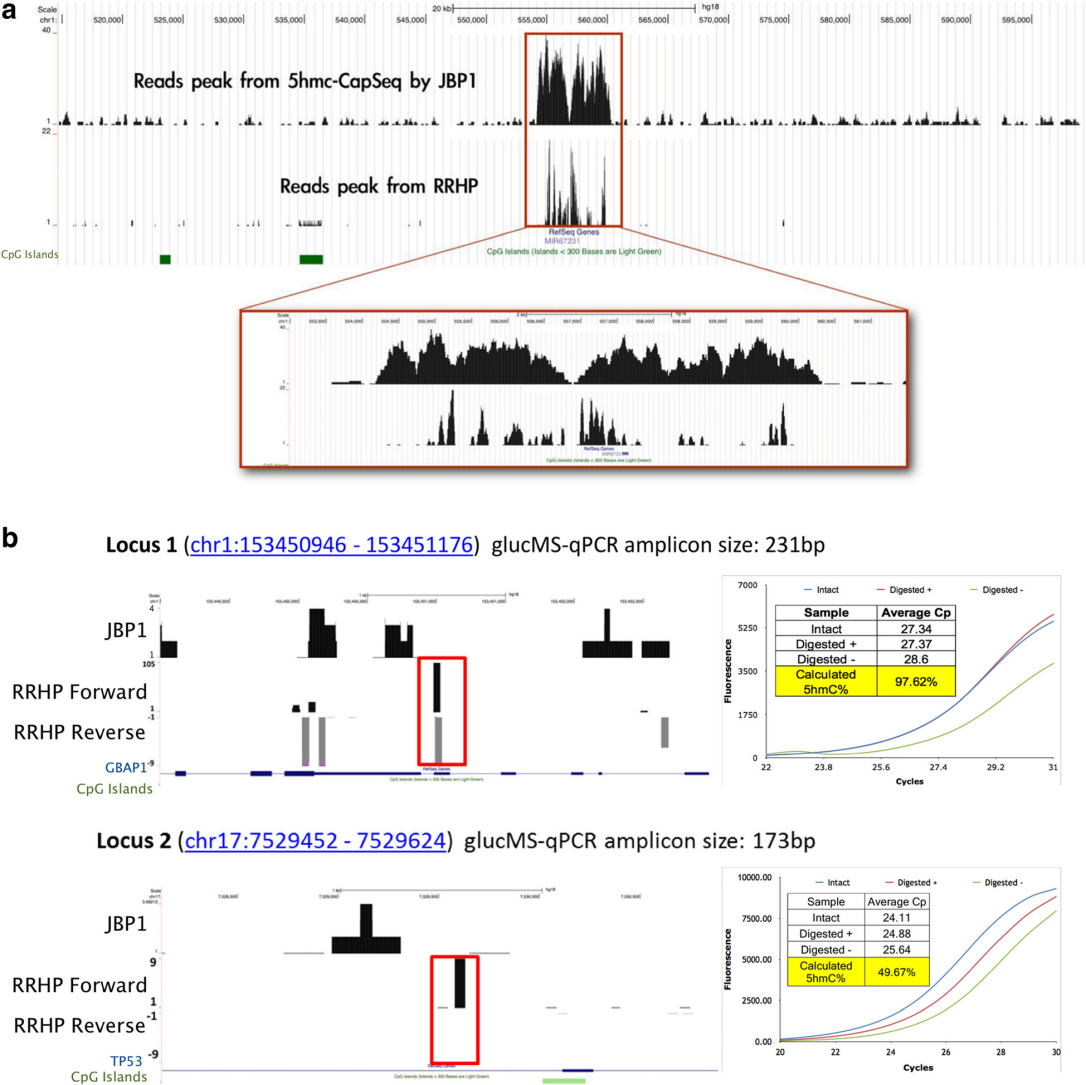
**Validating 5hmC sites from the RRHP assay by hMeDIP-Seq and glucMS-qPCR. (a)** Read peaks called by MACS program for hMeDIP-Seq and RRHP assay. **(b)** Locus specific validation by glucMS-qPCR at two 5hmC sites (highlighted by red block) in chromosome 1 (114 reads in RRHP) and chromosome 17 (9 reads in RRHP), which were not identified by hMeDIP-Seq. Briefly, samples were treated or mock-treated with β-GT and then digested with MspI. Following purification, the loci were amplified to determine resistance to MspI restriction conferred by glucosylation of the 5hmC position. The amplification profiles of the treatment (Digested +) and mock-treatment (Digested -) samples were compared to untreated, undigested gDNA (Intact). Digested + samples with a C_T_ closer to the intact sample indicates higher hydroxymethylation levels while a C_T_ more similar to the Digested - sample indicates less hydroxymethylation.

To further evaluate the reliability of the assay, we also performed genome-wide and locus-specific validations using a JBP-1-based enrichment assay [12] and a glucMS-qPCR assay [13], respectively. Both methods showed strong correlations with *de novo* RRHP discoveries. RRHP allowed more sensitive detection of low-abundant 5hmC sites, which cannot be resolved with enrichment approaches. We processed RRHP data with MACS program [14] to create a peak track analogous to that of enrichment sequencing. Overlay of the tracks revealed close similarity of peak distributions over 5hmC-containing regions (Figure 5A). Of the 4,000 peaks identified by the JBP-1 based hMeDIP-Seq, 40% overlapped with at least one 5hmC site profiled by RRHP. However, only 0.5% of the 5hmC sites detected by RRHP fell within the peak regions of hMeDIP-Seq. This indicates RRHP is more sensitive in identifying 5hmC sites although it is limited by the distribution of restriction sites. Importantly, RRHP peaks can be confidently called at regions that would have fallen at or below background with enrichment sequencing. For further validation using glucMS-qPCR, we selected two different loci not identified as a 5hmC peak in hMeDIP-Seq but were detected by RRHP with 114 and nine reads. Conveniently, the RRHP and glucMS-qPCR assays allowed for straightforward cross-validation due to the shared foundation of glucosyl-sensitive digestion reactivities. In the simplest embodiment of the method, each 5hmC positioned at a MspI junction was profiled by creating primers flanking the site. Genomic DNA was treated or mock-treated with β-GT, subjected to MspI digestion, and then amplified by qPCR to confirm glucosyl protection at the locus and to quantify the abundance of 5hmC at the position by ΔCt. The β-GT treated samples had a lower Ct than the mock-β-GT treated, indicating the presence of 5hmC at the two loci (Figure 5B). The 5hmC abundance was calculated to be approximately 97.62% and 49.67%, respectively, for these two sites using the formula mentioned in the method section.

### 5hmC profiling in breast and liver cancer samples by RRHP

**Figure 6:**
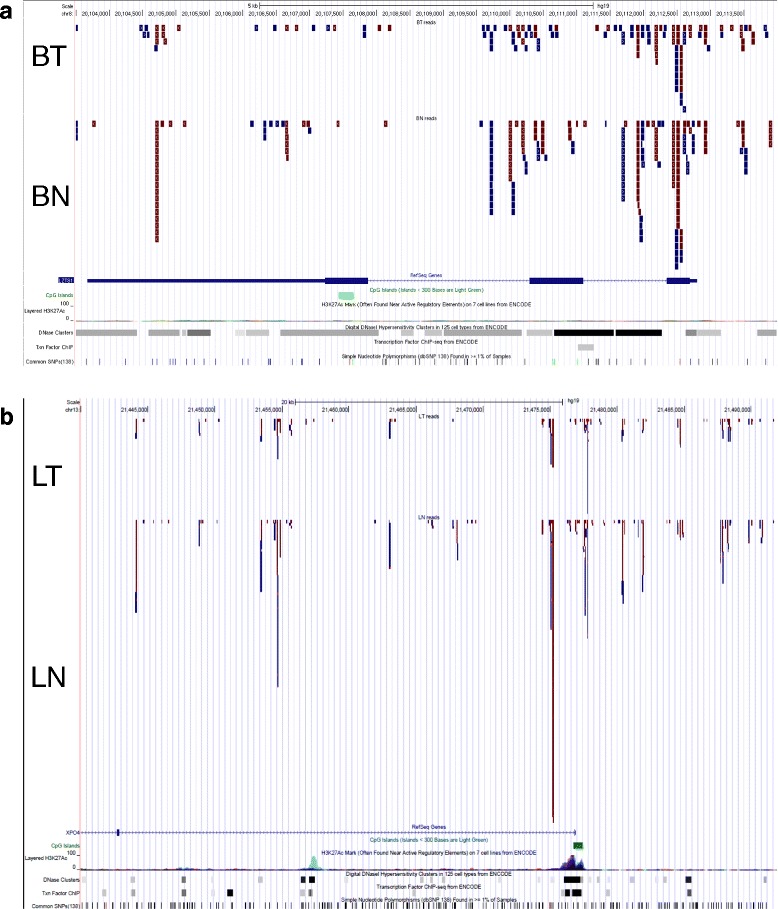
**Illustration of the RRHP genome browser read track for paired tumor samples. (a)** The read track for the LZTS1 locus shows that normal tissues had more 5hmC reads compared to the breast tumor (BT: breast tumor; BN: breast adjacent normal). **(b)** The XPO4 locus also had lower 5hmC reads in the liver tumor compared to the normal liver sample. (LT: liver tumor; BN: liver adjacent normal).

Previous studies on the role of 5hmC in cancer have shown loss of 5hmC is commonly associated with tumor development in both hematological diseases and solid tumors [15]. However, it is not clear whether the decrease of 5hmC is a result of global 5mC reduction, which is also a hallmark of tumorgenesis, or due to a different epigenetic regulation. Since those studies utilized either liquid chromatography-mass spectrometry (LC-MS/MS) or antibody-based immune dot blots and immunohistochemistry to measure 5hmC levels, 5hmC alternation with gene-level resolution cannot be detected. To gain a further understanding of how 5hmC loss is involved in tumor initiation or progression, it is necessary to identify the genes and signaling pathways that are regulated by changes in 5hmC. To this regard, we performed a pilot study for two types of paired solid tumor samples (breast and liver tumors as well as their adjacent normal tissues) using RRHP. In both cases, tumor and normal samples showed a very similar genomic distribution in terms of the total number of 5hmC sites detected. However, the 5hmC abundance at each CpG site was altered globally when we did a side-by-side comparison between the paired samples, which is reflected by the read counts change (as shown in Additional file 6). This result is in good agreement with previous observations that 5hmC levels tend to decrease in tumor samples [16,17]. GREAT analyses were also performed using the top 2,000 significantly different 5hmC sites between the paired tumor samples (Additional file 7). Genes enriched from breast tumor analysis were found to regulate processes such as osteoblast differentiation, gliogenesis, actin filament bundle assembly, and so on, which all have been proved to be associated with breast cancer metastasis previously [18-20]. On the other hand, genes enriched from the liver tumor analysis were more related to metabolic or biosynthetic regulation of sterol, steroid, ketone, lipid, fatty acid, and so on. The majority of genes with 5hmC reduction had been shown to be downregulated in hepatocellular carcinoma, indicating 5hmc loss might be used to transcriptionally inactivate certain tumor suppressors since early studies had shown that 5hmC, especially those located in gene bodies, were associated with transcriptional activity. As an example, we checked the 5hmC levels of two tumor suppressors: LZTS1 in breast cancer and XPO4 in liver cancer [21,22]. By examining 75 primary breast cancers and 12 normal breast tissues, Wielscher *et al.* had recently found that LZTS1 had significantly lower 5hmC content in tumors compared to normal breast tissues in the region between the 5′UTR to the second exon while no significant differences were observed for 5mC. Correspondingly, the *LZTS1* mRNA expression was reduced in the tumor samples, suggesting a strong influence of 5hmC on mRNA expression. Consistent with these results, the genome browser track from RRHP for the same region again showed a decrease of 5hmC in tumor in comparison to its adjacent normal (Figure 6a). This pattern was also observed for the liver tumor suppressor XPO4 (Figure 6b).

## Discussion

RRHP features a rapid workflow, avoids harsh chemical modification, and allows processing of DNA inputs as low as 100 ng. Also, RRHP is a positive display that eliminates the need for parallel subtractive sequencing as required by oxBS-Seq and does not need high sequencing depth to detect low 5hmC abundances as required by TAB-Seq, which needs an average of 26.5× read coverage to resolve a single 5hmC site with 20% abundance at a false discovery rate (FDR) <5%. Under the same FDR, we found that RRHP was able to confidently detect approximately one million 5hmC sites in human brain tissue with only 20 to 30 million reads. Since brain tissues have the highest 5hmC content compared to other tissues examined thus far, such sequencing depth should be sufficient for other tissues. However, it would still be helpful if a pilot library for an unknown sample was sequenced with a higher depth and then analyzed as a function of the total number of reads by down-sampling the data to determine the minimal sequencing depth required. This will enable the user to adjust the sequencing depth accordingly and make sequencing runs more cost-efficient. It is also worth noting that samples which need comparative analysis should be sequenced with the same depth or normalization is required. Typically, data for the same sample from runs with different sequencing depth can be normalized by the total number of reads. However, due to the positive display nature of RRHP, the total number of sequencing reads is not only associated with sequencing depth but also related to 5hmC abundance. Therefore, normalization by the total number of reads is inappropriate when comparing different samples since the number of sequencing reads is an indication of 5hmC abundance. Thus, 5hmC sites with housekeeping characteristics are needed to serve as an internal control to normalize samples. Alternatively, a spike-in control with various 5hmC levels would be helpful for normalization. Currently, we maintain the relative 5hmC abundance between samples by multiplexing samples with equal volume, rather than equal mass, for libraries prepared in parallel (that is, same DNA input, same purification, same amplification, and so on). For example, we multiplexed sample RRHP-MspI-2 with half the volume that of RRHP-MspI-1, and as expected, the total number of reads for RRHP-MspI-2 was half of RRHP-MspI-1 (Table 1). Without data normalization, the correlation between the two samples was 0.864, indicating little variation in the sample preparation (Figure 3b). Also, from our comparison of the paired tumor samples, our results were in agreement with previously published data that showed tumors have less 5hmC abundance.

The high sensitivity and low background associated with RRHP allows for both qualitative and relative quantitative descriptions of 5hmC at genomic loci, resulting in high correlation with previously described interrogative methods such as hMeDIP-Seq and glucMS-qPCR. The method detects 5hmC in a strand-specific fashion and can couple analyses of epigenetic modifications with genomic variation, such as SNP detection. When combined with RRBS data, the method allows for high resolution and direct correlation of 5mC and 5hmC positions. This system can be adapted for any platform (such as ion torrent PGM and so on) that utilizes adapterized libraries, and by using other glucose-sensitive restriction enzymes for fragmentation and library digestion, we can profile 5hmC sites in alternative CpG motifs as well as non-CpG contexts. This principle can also be applied in mapping other epigenetic modifications, such as 5fC, 5CaC and N6-methyladenine (6 mA) by using alternative restriction enzymes. In addition, three other enzyme-based methods were also recently developed for genome-wide 5hmC profiling including Aba-Seq, HELP-GT assay and HMST-Seq [23-25]. Aba-Seq utilizes a DNA-modification dependent restriction endonuclease, AbaSI, coupled with sequencing. AbaSI recognizes glucosylated 5hmC with high specificity and generates a double strand break 11-13 bp downstream of the recognition site. However, this enzyme prefers sites with two cytosines positioned symmetrically around the cleavage site, and the cleavage efficiency is lower when only one of the two cytosines is a glucosyl-5hmC. Putative 5hmC sites are indirectly deduced by checking for the presence of a cytosine at the expected distances from either side of the mapped cleavage sites. There are two major limits for this method: first, certain 5hmC sites may not be detected due to the low cleavage efficiency caused by the absence of symmetric pattern of the recognition site. Second, it causes assignment ambiguity to the exact cytosine in categories which has 2CGs or 2CHs at the symmetric recognition site, and these sites account for 13% of all identified cleavage sites, according to the authors. In addition, Aba-Seq requires a much higher sequencing depth; over 200 million reads is needed for an Aba-Seq library, making it not cost competitive to RRHP. The other two assays, HELP-GT and HMST-Seq, are more similar to RRHP in terms of the restriction enzyme used and the genomic coverage, but both of them are negative display methods and require subtractive sequencing. In other words, two libraries for each sample have to be sequenced in order to infer the 5hmC status for a CpG site, and it is challenging to normalize the data for subtraction given a variety of factors which may affect the read counts and distribution between the two libraries. Lastly, both HELP-GT and HMST-Seq assays have a very complicated workflow which requires multiple enzymatic digestions, sequential adapterization, bead capture or *in vitro* transcription, making it not ideal for samples with low DNA input.

## Conclusions

Here we present a novel approach, RRHP, for genome-wide profiling of 5hmC, which exploits β-glucosyltransferase (β-GT) to inhibit restriction digestion at adapters ligated to a genomic library, such that only fragments presenting glucosylated 5hmC residues at adapter junctions will be amplified and sequenced. This assay profiles 5hmC sites with single-base resolution in a strand-specific fashion. When combined with existing RRBS data, it allows for simultaneous comparison of 5mC and 5hmC at a specific site. We find that this assay is a robust and cost-efficient tool for profiling 5hmC across the genome.

## Methods

### Adapter design and construction

P5CCG and P7CG adapter pairs were constructed in a manner that allowed for 5′-CG overhangs instead of the standard 5′-T overhangs of the Illumina TruSeq P5 and P7 adapters. In the P5CCG adapter pair, CCGG is retained at the junction following ligation to a library fragment while in the P7CG adapter pair, the CCGG junction becomes TCGG at ligation and is no longer sensitive to HpaII or MspI restriction. For both adapter pairs, two long oligos were hybridized with their respective complementary short oligos at 50 μM with a slow ramp-down (0.1°C/s) from 95°C to 12°C in oligo hybridization buffer (50 mM NaCl, 1 mM Tris-HCl pH 8.0, 100 μM EDTA). The P5 adapter pair was prepared from HPLC-purified oligos (IDT): 5′-ACACTCTTTCCCTACACGACGCTCTTCCGATCTC-3′ (long) and 5′-CGGAGATCGGAAGAG-3ddC -3′ (short). The P7 adapter pair was prepared from the oligos: 5′-GTGACTGGAGTTCAGACGTGTGCTCTTCCGATCT-3′ (long) and 5′- CGAGATCGGAAGAG-3ddC -3′ (short).

### RRHP library construction and sequencing

Genomic DNA from human male cerebellum or tumor and adjacent normal tissue was purified via phenol:chloroform extraction and digested for 8 h at 37°C with 20 U MspI (NEB). Following digestion, the enzyme was inactivated at 65°C for 15 min and fragmented gDNA was purified using the DNA Clean and Concentrator kit (Zymo Research). For adapterization, 100 or 500 ng of fragmented DNA was ligated overnight at 16°C with 400 U T4 DNA Ligase (NEB) and 500 nM modified P5 and P7 adapters (IDT), such that the CCGG junction was retained at the P5 adapter and destroyed in the P7 adapter (see ‘Adapter construction’ for sequence detail). After overnight incubation, adapters were extended with 2 U GoTaq (Promega) and 500 μM dNTPs (Zymo Research) at 72°C for 30 min. Following extension, adapterized libraries were purified with the DNA Clean and Concentrator kit. Adapterized libraries were glucosylated with 10 U β-GT (Zymo Research) and 100 nM UDPG at 37°C for 4 h. For negative control reactions, β-GT was omitted from the incubation. To all of the reactions, 20 U of MspI or HpaII were then added and incubated at 37°C overnight. After digestion, an additional 20 U of MspI or HpaII were added and allowed to incubate for an additional 1 h. Enzymes were heat inactivated at 65°C and libraries were purified with the DNA Clean and Concentrator kit. Purified libraries were then loaded in a 2.5% (w/v) 50:50 NuSieve:agarose gel and electrophoresed. Size-selected libraries were cut from 110 to 500 bp and purified with the Zymoclean Gel DNA Recovery Kit (Zymo Research). Finalized libraries were then amplified with 500 nM P5/P7 barcoding primers (IDT) in OneTaq 2X Master Mix (NEB) with the thermal profile: 94°C for 30 s, 58°C for 30 s, and 68°C for 30 s, repeated for 10 cycles. Amplifications were sampled for visualization on a 2% agarose and purified with the DNA Clean and Concentrator kit. For sequencing, equal volumes of each amplified library were pooled and diluted to 8 pM for 50 bp singleton reads on the Illumina HiSeq 2000 (Illumina).

### Bioinformatic processing and statistical analyses

Sequencing reads from the RRHP assay were first processed to trim off low quality bases and the P7CG adapter at the 3′ end of the reads and then aligned to the hg18 build of the human genome using Bowtie0.12.8 and its default parameters with --best. Aligned reads with CCGG tag at 5′ end were counted. The correlation analysis between the different RRHP libraries were performed by comparing the presence of the tagged reads at each profiled MspI site, and the Pearson’s coefficient was calculated accordingly. The reads for RRBS library were processed as previously reported. Gene ontology analysis was performed using the Genomic Regions Enrichment of Annotations Tool (GREAT) [11].

### JBP-1-mediated enrichment sequencing library preparation and analysis

The enrichment sequencing libraries were prepared from 1 ug gDNA fragmented with dsDNA Shearase (Zymo Research). Fragments were then A-tailed with Klenow exo- fragment (NEB) and ligated to adapters per standard Illumina library preparation protocols. Libraries were glucosylated with β-GT and enriched via incubation with immobilized JBP-1 using the Quest 5hmC DNA Enrichment Kit (Zymo Research) per the manufacturer’s protocol. Libraries were subjected to limited amplification, purified, and sequenced on the Genome Analyzer IIX platform (Illumina). Resulting reads were trimmed for adapters, aligned to hg18 with Bowtie, and analyzed for enrichment peak calling in MACS.

### glucMS-qPCR validation of de novo 5hmC discovery loci

A total of 100 ng of gDNA from the same human male cerebellum sample was glucosylated with 10 U β-GT (Zymo Research) and 100 nM UDPG (Zymo Research) or mock-treated without enzyme at 37°C for 2 h. To the same reactions, 20 U of MspI (NEB) were added and incubated for an additional 2 h. Following heat inactivation at 65°C for 15 min, reactions were purified with the DNA Clean and Concentrator kit (Zymo Research) and quantified. 10 ng of each treatment group was utilized for qPCR in triplicate with QuestTaq qPCR Master Mix (Zymo Research) and 200 nM primers (IDT). Reactions were amplified on a CFX96 cycler (Bio-Rad) with the thermal profile: 95°C for 3 min, 40 cycles of 95°C for 30 s, 60°C for 20 s, 72°C for 20 s, and then a final extension at 72°C for 1 min before a 4°C hold. All amplifications were then subjected to melt curve analysis to ensure specific amplification and identity. Cp values were averaged from three technical replicates for each treatment and 5hmC% was calculated using the equation ((Digested -)-(Digested +) / (Digested -) - (Intact)) × 100%.

Primers for glucMS-qPCR Validation:

- Locus 1 Chr1 : 153450946 - 153451176
- Fwd: 5′ CTTCAGCCCACTTCCCAGAC
- Rev: 5′ GTGGGTGGGCGACTTCTTAG
- Locus 2 Chr17: 7529452 - 7529624
- Fwd: 5′ AAGGACAGAAGCCCGACAAA
- Rev: 5′ CAGCTATTCGGGAGGGTGAG

### Data access

The RRHP sequencing data from this study have been submitted to NCBI’s Gene Express Omnibus (GEO) under accession number GSE49546.

## Additional files

Additional file 1:
**The frequency distribution of the 5hmC sites with different read coverage profiled by RRHP assay.**
Additional file 2:
**Pairwise comparison between libraries prepared with different inputs.** (a) Venn diagrams show number of 5hmC sites in RRHP-MspI-1 and RRHP-MspI-3 with 725,464 sites in common and 154,226 unique sites in RRHP-MspI-1 and 99,237 unique sites under the condition of five reads cutoff. (b) Plotting Pearson’s correlation coefficient from the comparison between RRHP-MspI-1 and RRHP-MspI-3 against sequencing read cutoff. Additional file 3:
**Integrative representation of RRBS and RRHP data for** ***RASSP1*** **gene in UCSC genome browser.** CpG sites with low methylation in RRBS show no or few reads in RRHP. Most of the RRHP reads were found in the gene promoter of RASSP1, which also overlap with H3K4me1 and H3K4me3 modification as well as the DNaseI hypersensitive cluster in that region (highlighted by green frame). Interestingly the reverse strand (coding stand) has higher hydroxymethylation than the forward strand (non-coding strand) as indicated by the number of the reads in each strand (arrows in the reads indicate either forward or reverse strand). Red: non methylation, yellow: methylation (The number next to each CpG site indicates number of methylated reads in total reads; for example, 4/34 means four methylated read in total 34 reads covering that CpG site). Additional file 4:
**Breakdown of 5hmC sites profiled by RRHP into specific annotated genomic elements and binding locations.** All annotations were obtained from UCSC Genome Browser (hg18). CpG islands were directly obtained from CpG Islands track. 5′UTR, 3′UTR, promoter, exons, and introns were based on RefSeq Genes track. Promoter was defined as 1 kb upstream and downstream of TSS. High-CpG promoters (HCPs), weak CpG islands or intermediate-CpG promoters (ICPs), and sequences with no local enrichment of CpGs or low-CpG promoters (LCPs) were calculated based on Weber *et al*. [26]. Coordinates for regions of 7× regulatory potential or regions (average score >0.5) conserved in human, chimpanzee, macaque, mouse, rat, dog, and cow were obtained from King *et al*. [27]. Histone ChIP-chip binding data was H1ES H3K4me3 and H1ES H3K27me3 tables from Broad Histone track. Bivalent regions were areas that overlapped with both H3K4me3 and H3K27me3 peaks. RRHP regions overlapping with H3K4me3 peaks only were called H3K4me3; regions overlapping with H3K27me3 peaks only were called H3K27me3. Additional file 5:
**Illustration of RRHP data in UCSC genome browser.** The strandedness of each CpG site in the RRHP-CpG track is indicated by blue (forward strand) and red (reverse strand), and the number next to each CpG indicates the read coverage. The same color code applies in the RRHP read track with a letter indicating the SNPs on each strand. Additional file 6:
**Box plot shows read count distribution for all the common sites between the paired tumors.**
Additional file 7:
**GREAT analysis for the top 2,000 5hmC sites between paired tumor and normal sample.** The input of GREAT is the combination of top 1,000 sites from intersection data (all sites must be non-zero) and top 1,000 sites form symmetric difference sites (one sites must be zero). No site selection and normalization were applied to the above data set. The ranking criteria are absolute values of log ratio (tumor count/normal count) from large to small.
