# Supplementary figures and images for: RRHP: a tag-based approach for 5-hydroxymethylcytosine mapping at single-site resolution
Source: Genome Biol. 2014 Sep 24;15(9):456. doi: 10.1186/s13059-014-0456-5 (PMC4212096; doi:10.1186/s13059-014-0456-5)

## Slide 1
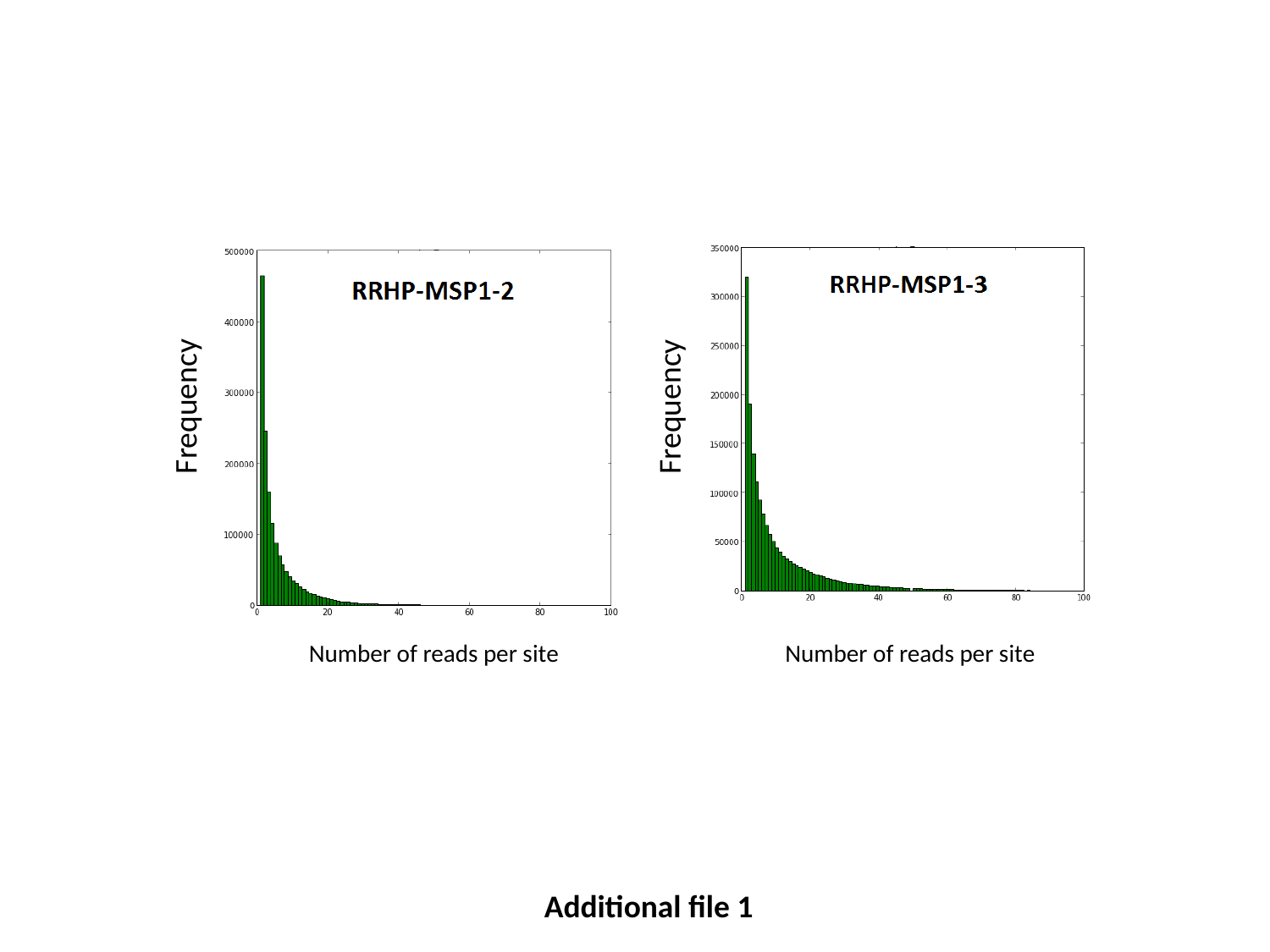

Frequency
Frequency
Number of reads per site
Number of reads per site
Additional file 1

Supplement: Additional file 1: — The frequency distribution of the 5hmC sites with different read coverage profiled by RRHP assay. [file 13059_2014_456_MOESM1_ESM.pptx]

## Slide 1
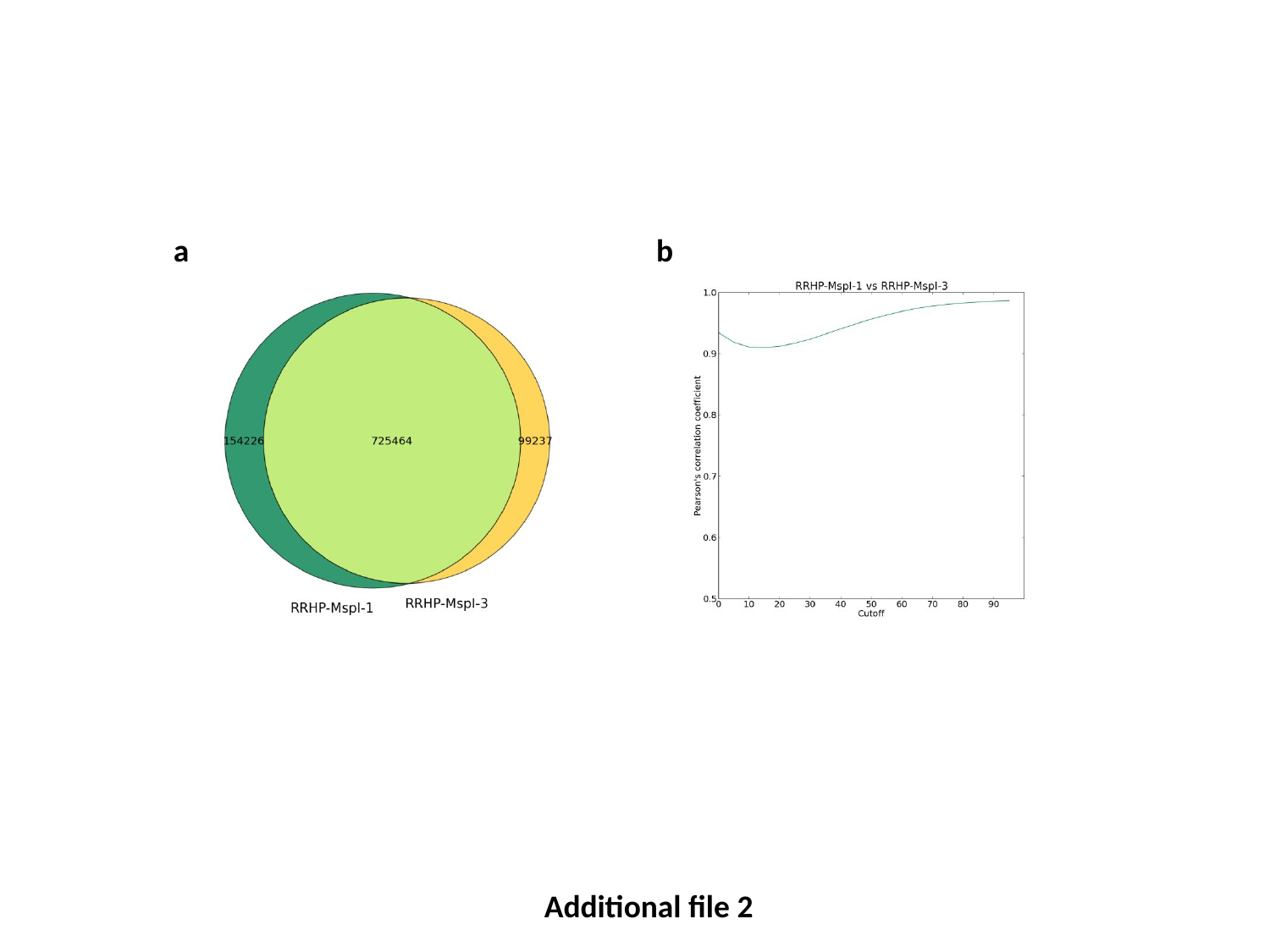

a
b
Additional file 2

Supplement: Additional file 2: — Pairwise comparison between libraries prepared with different inputs. (a) Venn diagrams show number of 5hmC sites in RRHP-MspI-1 and RRHP-MspI-3 with 725,464 sites in common and 154,226 unique sites in RRHP-MspI-1 and 99,237 unique sites under the condition of five reads cutoff. (b) Plotting Pearson’s correlation coefficient from the comparison between RRHP-MspI-1 and RRHP-MspI-3 against sequencing read cutoff. [file 13059_2014_456_MOESM2_ESM.pptx]

## Slide 1
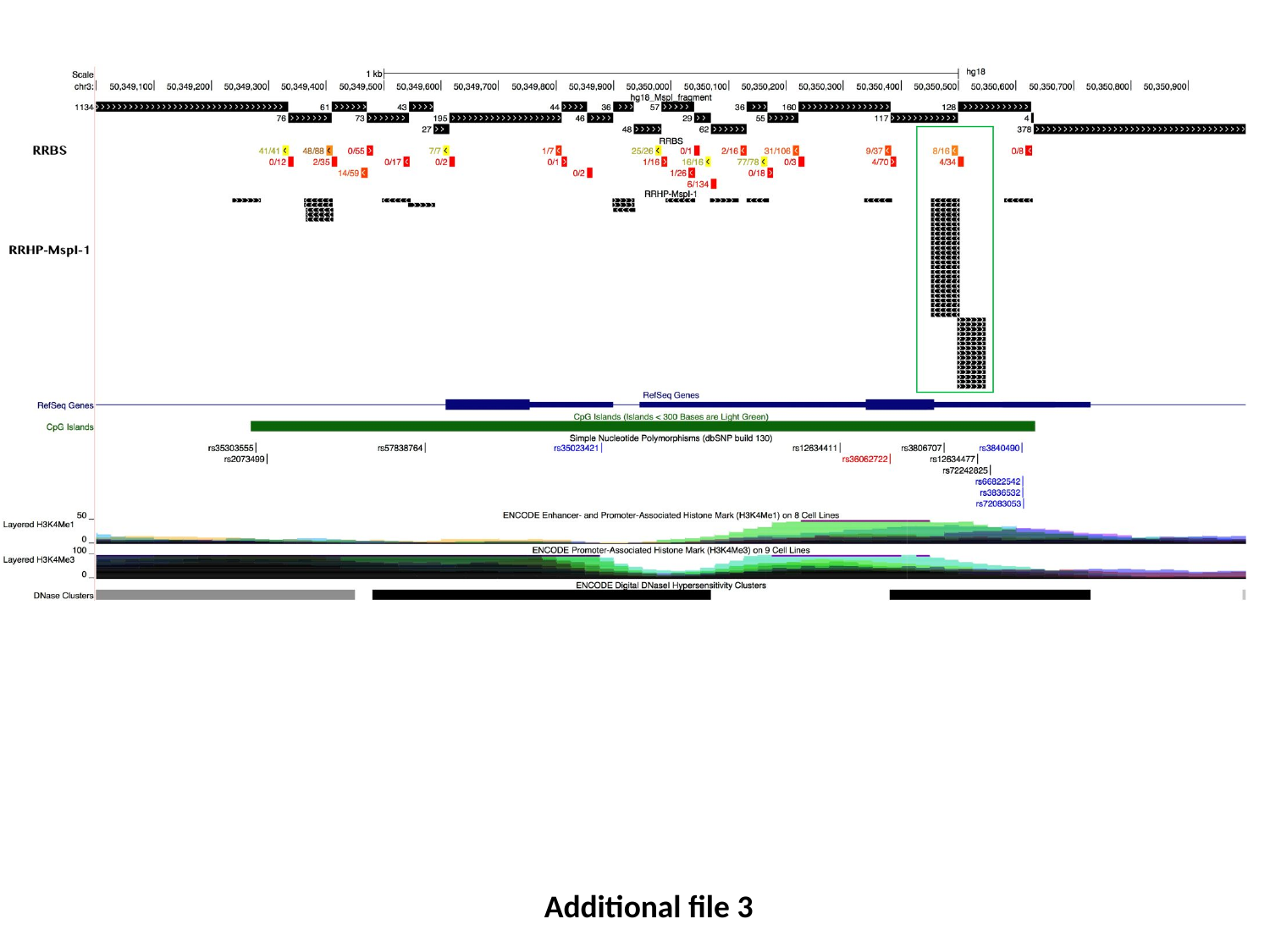

Additional file 3

Supplement: Additional file 3: — Integrative representation of RRBS and RRHP data for RASSP1 gene in UCSC genome browser. CpG sites with low methylation in RRBS show no or few reads in RRHP. Most of the RRHP reads were found in the gene promoter of RASSP1, which also overlap with H3K4me1 and H3K4me3 modification as well as the DNaseI hypersensitive cluster in that region (highlighted by green frame). Interestingly the reverse strand (coding stand) has higher hydroxymethylation than the forward strand (non-coding strand) as indicated by the number of the reads in each strand (arrows in the reads indicate either forward or reverse strand). Red: non methylation, yellow: methylation (The number next to each CpG site indicates number of methylated reads in total reads; for example, 4/34 means four methylated read in total 34 reads covering that CpG site). [file 13059_2014_456_MOESM3_ESM.pptx]

## Slide 1
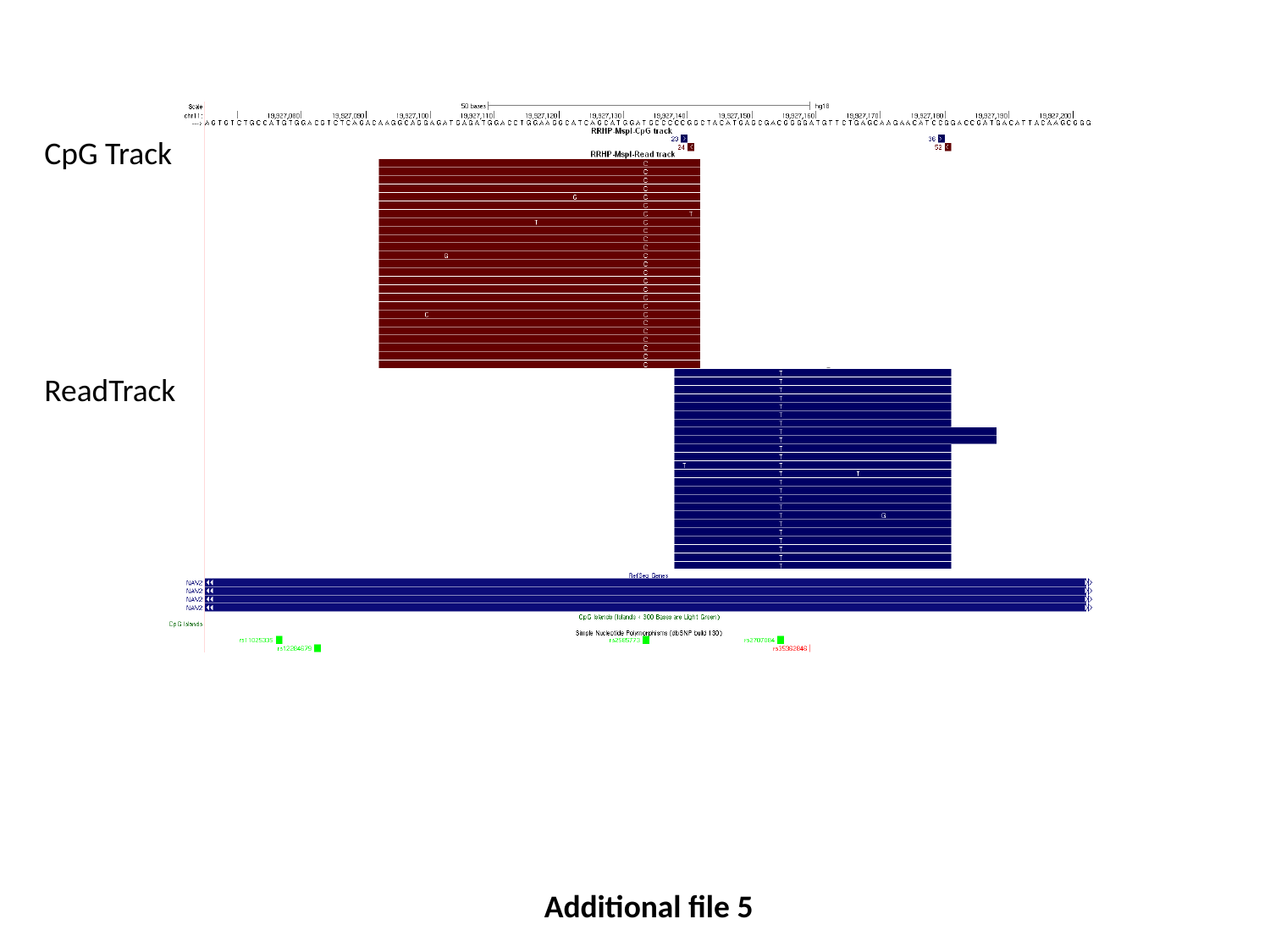

CpG Track
ReadTrack
Additional file 5

Supplement: Additional file 5: — Illustration of RRHP data in UCSC genome browser. The strandedness of each CpG site in the RRHP-CpG track is indicated by blue (forward strand) and red (reverse strand), and the number next to each CpG indicates the read coverage. The same color code applies in the RRHP read track with a letter indicating the SNPs on each strand. [file 13059_2014_456_MOESM5_ESM.pptx]

## Slide 1
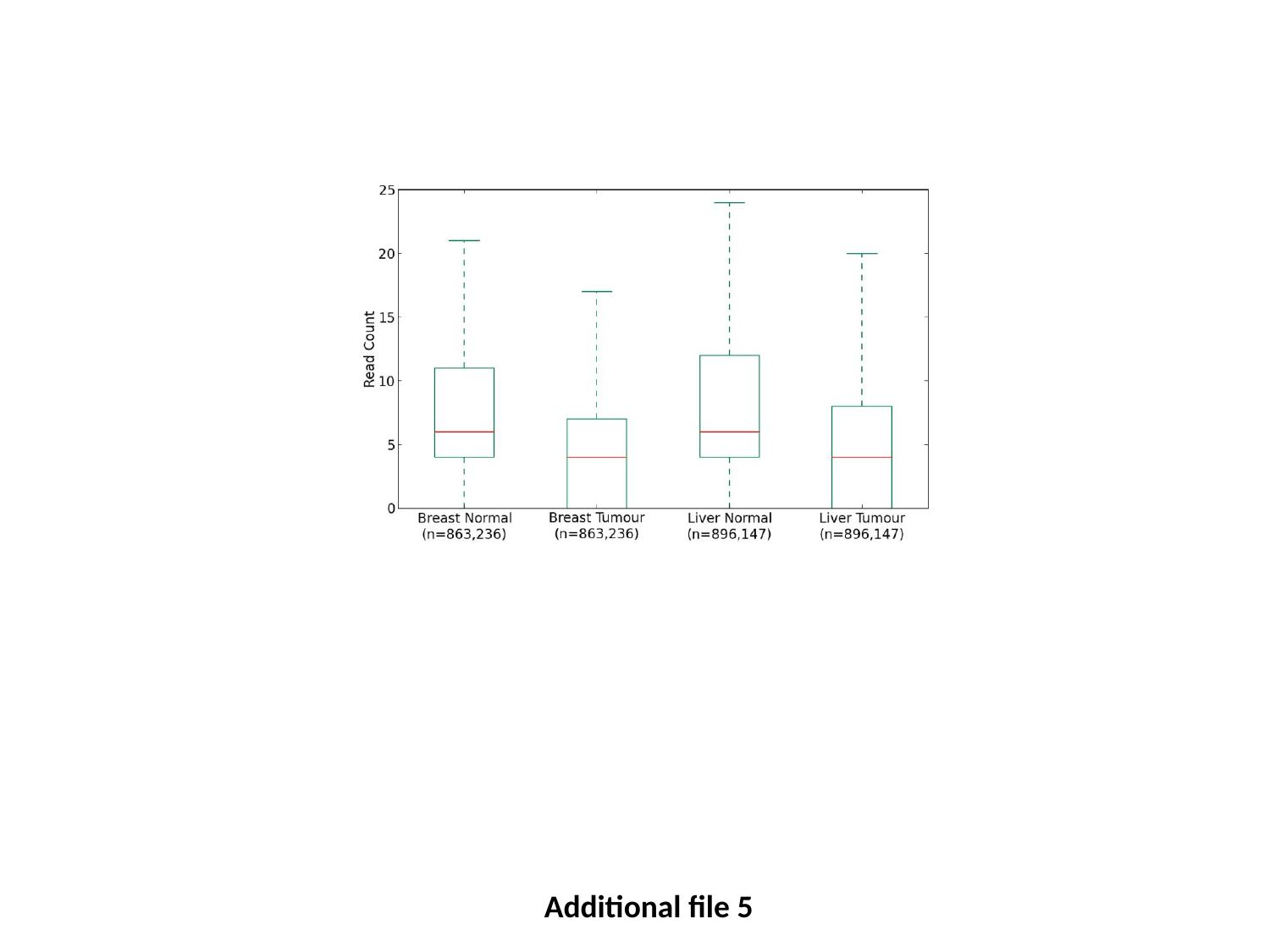

Additional file 5

Supplement: Additional file 6: — Box plot shows read count distribution for all the common sites between the paired tumors. [file 13059_2014_456_MOESM6_ESM.pptx]
