# Supplementary material for: RRHP: a tag-based approach for 5-hydroxymethylcytosine mapping at single-site resolution
Source: Genome Biol. 2014 Sep 24;15(9):456. doi: 10.1186/s13059-014-0456-5 (PMC4212096; doi:10.1186/s13059-014-0456-5)
Supplement: Additional file 4: — Breakdown of 5hmC sites profiled by RRHP into specific annotated genomic elements and binding locations. All annotations were obtained from UCSC Genome Browser (hg18). CpG islands were directly obtained from CpG Islands track. 5′UTR, 3′UTR, promoter, exons, and introns were based on RefSeq Genes track. Promoter was defined as 1 kb upstream and downstream of TSS. High-CpG promoters (HCPs), weak CpG islands or intermediate-CpG promoters (ICPs), and sequences with no local enrichment of CpGs or low-CpG promoters (LCPs) were calculated based on Weber et al. [26]. Coordinates for regions of 7× regulatory potential or regions (average score >0.5) conserved in human, chimpanzee, macaque, mouse, rat, dog, and cow were obtained from King et al. [27]. Histone ChIP-chip binding data was H1ES H3K4me3 and H1ES H3K27me3 tables from Broad Histone track. Bivalent regions were areas that overlapped with both H3K4me3 and H3K27me3 peaks. RRHP regions overlapping with H3K4me3 peaks only were called H3K4me3; regions overlapping with H3K27me3 peaks only were called H3K27me3. [file 13059_2014_456_MOESM4_ESM.docx]

|  | RRHP-MspI-1  (0.5 µg) | | RRHP-MspI-2  (0.5 µg) | | RRHP-MspI-3  (0.1 µg) | |
| --- | --- | --- | --- | --- | --- | --- |
| Total | 1737993 |  | 1550791 |  | 1674080 |  |
| CpG island | 171874 | 10% | 175580 | 11% | 144290 | 9% |
| Promoter | 137374 | 8% | 146830 | 10% | 114982 | 7% |
| 5' UTR | 211557 | 12% | 198467 | 13% | 197653 | 12% |
| exon | 157468 | 9% | 146740 | 10% | 145542 | 9% |
| intron | 782758 | 45% | 699360 | 45% | 756503 | 45% |
| 3' UTR | 93850 | 5% | 83242 | 5% | 90372 | 5% |
| bivalent | 126206 | 7% | 122290 | 8% | 108091 | 7% |
| H3K4me3 | 201398 | 12% | 206782 | 13% | 171203 | 10% |
| H3K27me3 | 513438 | 30% | 457022 | 30% | 485723 | 29% |
| HCP | 101578 | 6% | 115454 | 7% | 80867 | 5% |
| ICP | 23918 | 1% | 21404 | 1% | 22428 | 1% |
| LCP | 12879 | 1% | 10925 | 1% | 12557 | 1% |
| 7X Regulatory  potential | 684996 | 39% | 612015 | 40% | 645167 | 39% |

**Additional file 4**
